# COVID-19 Vaccine and Active Infection Associated Neuropsychiatric Manifestations in an Adolescent: A Literature Review

**DOI:** 10.1192/bjo.2025.10244

**Published:** 2025-06-20

**Authors:** Neel Vyas

**Affiliations:** Leeds Teaching Hospitals NHS Trust, Leeds, United Kingdom

## Abstract

**Aims:** The aim of this study is to evaluate the literature for COVID-19 induced psychosis in a child and adolescent population.

**Methods:** This review included a comprehensive literature search across major databases, including MEDLINE, EMBASE, PsycINFO, and the Cochrane Library. The inclusion criteria encompassed case reports, case series, longitudinal studies, and observational studies involving children or adolescents (under 18 years) who had a diagnosis of COVID-19 or a recent history of COVID-19 vaccination and presented with features of psychosis. Data extraction focused on key demographic information, such as age, gender, and past medical and psychiatric history. Additionally, details regarding COVID-19 treatment and the management of psychosis were recorded.

**Results:** Eight studies included patients with a history of COVID-19 infection or vaccination who subsequently presented with psychosis. Five were male (62.5%), and three were female (37.5%). COVID-19 infection was confirmed by PCR (50%), antibody tests (25%), positive viral swabs (12.5%), or positive lateral flow tests (12.5%). Most patients were managed conservatively for COVID-19 (50%), while others received steroids (37.5%) or antibiotics (12.5%). Antipsychotic medication was the primary treatment for psychosis in most cases (75%), with some patients also receiving lithium (12.5%), SSRIs (12.5%), or benzodiazepines (12.5%). Regarding prior psychiatric history, 62.5% of patients had no previous psychiatric diagnoses. However, 25% had a history of learning disabilities, and 12.5% had a history of depressive disorder and illicit drug use.

**Conclusion:** Neuropsychiatric manifestations of COVID-19 in adolescents are less frequently reported compared with adults. Common presentations include insomnia, mood disorders, and, less commonly, psychosis. The precise mechanisms underlying these neuropsychiatric complications remain unclear, but they are hypothesized to be linked to an exaggerated immune response, including cytokine storms, triggered by the infection. This review highlights the need for further research and the development of specific management guidelines for these patients within this population.

